# Managing periprosthetic fractures – a review of the hub and spoke model

**DOI:** 10.1051/sicotj/2022001

**Published:** 2022-01-18

**Authors:** Srikanth Mudiganty, Luke Hughes, Qaisar Choudry, Awais Bokhari

**Affiliations:** 1 Senior Clinical Fellow, Trauma and Orthopaedics, Barts Health NHS Trust E1 1FR London UK; 2 Specialist Registrar, Trauma and Orthopaedics, East Lancashire NHS Hospitals Trust BB2 3HH Blackburn UK; 3 Consultant, Trauma and Orthopaedics, East Lancashire NHS Hospitals Trust BB2 3HH Blackburn UK

**Keywords:** Periprosthetic fractures, Unified classification system, Hub, Spoke

## Abstract

*Introduction*: Periprosthetic fractures are associated with significant morbidity and mortality. The “hub and spoke model” consists of a central organisation (the hub) and a series of secondary units (the spokes). This study reviews the presentation, management, and outcomes of periprosthetic fractures at a large general district hospital, the Royal Blackburn Hospital. *Methods*: A retrospective data analysis for patients presenting with periprosthetic fractures from a single general district hospital between January 2011 and December 2020. Details recorded were patient demographics, primary arthroplasty procedure, fracture management, ASA grade, morbidity and mortality, and Unified Classification System for Periprosthetic Fractures (UCSPF). *Results*: With 229 periprosthetic fractures, the number tripled in 2020 that admitted in 2011. The mean age was 78.6 years (range 33–100), 151 were females. Seventy-five percent of the fractures were managed locally, while 25% a referral to the higher specialist centre was sort. Of the 57 referrals, 50 were transferred to the hub, 5 were operated on locally, and 2 were managed non-operatively. Higher-level care transfer resulted in a delayed definitive treatment (4.8 versus 12 days, *p* = 0.001). About 94.4% of patients treated locally had a favourable outcome versus 92% of patients treated at the hub hospital. Cumulative mortality rates for the two sites were comparable. *Discussion*: Most of the patients presenting to the local spoke hospital with periprosthetic fractures were managed in house. For this practice to be preserved, there is a need for future planning, such as maintaining an appropriate skill mix at spoke units. Discussion between specialists at the hub and spoke hospitals reduced patient transfer by 14%.

## Introduction

The number of joint arthroplasty procedures are increasing year on year. The UK’s national joint registry recorded 208,318 in 2020 [1] as compared to 201,534 in 2015 [2]; an increase of 6758 (3.67%). These procedures have proved highly successful, with relief of pain, restoration of mobility, and improved quality of life. However, with patients living longer and with increasing fragility, the burden of periprosthetic fractures is also increasing [3]. Posing a major technical challenge to the surgeon and risk to the patient, data from the UK’s National Joint Registry reveals that surgery for periprosthetic fracture is associated with a higher mortality rate than revision for infection, dislocation, or aseptic loosening [4]. Patient outcomes following these complex revision surgeries can be highly variable, with many studies showing patient outcomes are higher when surgery is performed at high volume centres [5]. Service development necessitates detailed financial and resource planning, as periprosthetic fracture management comes at a substantial cost to the healthcare system. One 10-year study from a UK teaching hospital determined that the average cost of treatment for periprosthetic hip fractures was £23,469 (range £615–£223,000; median £18,031) [6].

The “hub and spoke model” sets up an infrastructure to pool resources and expertise. This model consists of a central organisation (the hub), which has the professional skill mix and equipment to manage all potential cases, and a series of secondary units (the spokes), which have comparatively limited services [7]. A well-designed “hub and spoke model” has numerous advantages, ensuring that patients’ needs are met while conserving and appropriately directing the allocation of resources and ensuring adequate nationwide coverage. Concentrating exposure can act to consolidate experience, facilitate learning, and optimise outcomes. MDT meetings between specialists at the hub centre and spoke units provide support and empower spoke units to undertake and manage simple periprosthetic fracture cases, thus easing the burden upon the hub [8]. Challenges associated with the set-up of a hub and spoke network include determining where appropriate to have the central hub. Often this will be the hospital with the greatest volume of cases and consultant expertise. However, those directing service developments must investigate local expertise, access to other services, multidisciplinary capabilities, academic productivity, and transport links to potential spoke units. With an increased transfer of patients to specialist centres, there are risks of congestion at the hub [1]. This must be anticipated with appropriate reallocation of resources. It is also important to recognise and address potential dissatisfaction at the spoke units, a consequence of perceived loss of autonomy in clinical decision making and delivery of local services. This requires that local healthcare professionals be included in service development decisions and educated on the perceived benefits [10].

Despite being clearly defined in the literature, few general district hospitals have published their experience of the hub and spoke model for periprosthetic fracture management. This study aims to detail the evolving workload, outline patient characteristics, review the management, and outcomes of periprosthetic fractures presenting to a large general district hospital, the Royal Blackburn Hospital. The authors include a critical evaluation of the regional hub and spoke network and advise future service development.

## Methods

This study took the form of a retrospective case series, with an analysis of data for all patients presenting with periprosthetic fractures to a single general district hospital between January 2011 and December 2020. Patient notes and the hospital’s online databases were reviewed to determine patient demographics (hospital identification number, age, gender, ASA grade), details of the primary procedure (date, joint, and implant), subsequent fracture presentation (date and mechanism of trauma), management (method, date, and location of definitive management), radiological outcomes (evidence of fracture healing), review of complications (morbidity and mortality). All presentations were classified in accordance with the Unified Classification System for Periprosthetic Fractures (UCSPF) [11].

## Results

**Figure 1:**
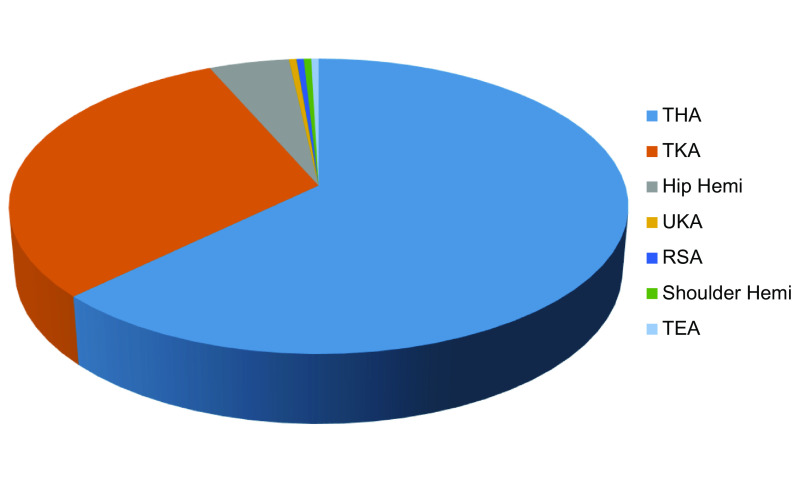
Location of periprosthetic fractures.

Over the 10-year study period, 229 periprosthetic fractures were presented to the spoke unit. The burden of disease was found to be increasing with the number of fractures in 2020, triple that admitted in 2011. The mean age was 78.6 years (range 33–100), with 151 females and 78 males. More than two-thirds of the patients were ASA grade 3 or above. Total hip arthroplasties (THA) accounted for 62.89%, while total knee arthroplasties (TKA) accounted for 30.6% of the cases. Eleven cases followed a hip hemiarthroplasty, while unicondylar knee arthroplasty, reverse shoulder arthroplasty, shoulder hemiarthroplasty, and total elbow arthroplasty accounted for 1 case each (Figure 1). Approximately 60% of THA’s were cemented, and 85% of TKA’s were cruciate retaining. According to the UCSPF, the majority of the fractures were IV.3.B2 (30.6%), followed by IV.3.B1 (17.9%), and V.3.B1 (15.7%). Further analysis of the total cohort revealed 32.75% were managed non-operatively, 36.25% of the fractures were treated with open reduction and internal fixation, and 27% underwent a revision. The mean time to surgery was 6.06 days (range 1–24).

**Figure 2:**
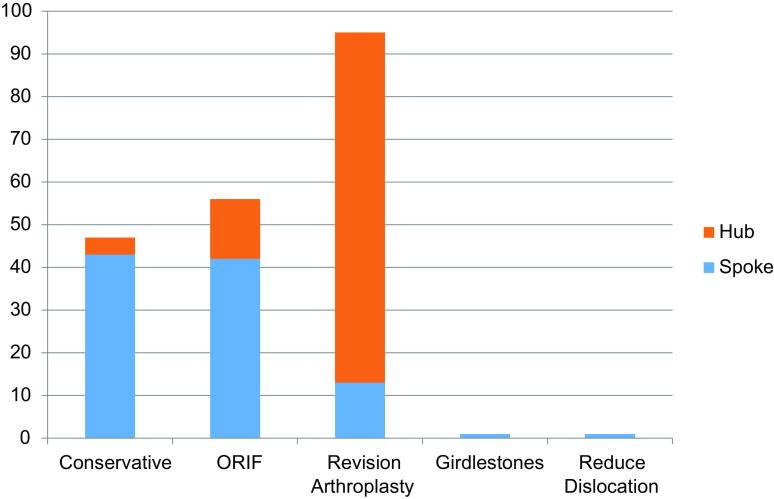
Treatment modalities at hub versus spoke.

Seventy five percent of the fractures were managed locally, while in 25%, a referral for input from the higher specialist centre was made. Of the 57 referrals, 50 were transferred to the hub, 5 were operated on locally following advice received from the hub, and 2 were managed non-operatively. A comparison between patients treated in house and those transferred to the hub is detailed in Table 1. Patients managed locally were on average older (79.9 versus 75.5 yrs, *p* = 0.008). However, patients were comparable for gender distribution and ASA grade. All type A and type D together with the vast majority of type B1 and type C fractures were managed locally. Of those patients transferred to the hub, 80% were type B2, wherein the implant is loose, yet there is adequate bone stock. Of those patients managed at the local spoke hospital, 43% were with conservative measures, 42% with open reduction internal fixation, 13% with revision arthroplasty, while 1% underwent a girdlestone’s procedure to remove all metal or received a general anaesthetic to reduce an associated joint dislocation in isolation. By comparison, of the 50 transferred to hub hospital, 82% underwent a revision arthroplasty procedure, 14% underwent open reduction internal fixation, while just 4% were managed non-operatively (Figure 2).

**Table 1:** Comparison between patients treated inhouse (Spoke) and those transferred to the Hub.

	Hub		Spoke		
Cases managed	178		50		
Mean patient age	79.9		75.3		*p* = 0.008
ASA	1	5	1	1	
	2	46	2	15	
	3	126	3	34	
	4	1	4	0	
Fracture classification	A	11	A	0	
	B1	78	B1	6	
	B2	39	B2	40	
	B3	2	B3	2	
	C	42	C	2	
	D	5	D	0	
	E	0	E	0	
	F	1	F	0	
Time to surgery (days)	Mean **4.8** (range 1 – 15; median 3.5)		Mean **12.0** (range 2–60; median 10)		*p* = 0.001
30-day mortality	4		0		*p* = 0.588
Further revision	4		4		

A transfer for higher-level care resulted in a delay for definitive treatment (4.8 versus 12 days, *p* = 0.001). Despite delays, outcomes in both groups were good. Considering all treatment modalities – 94.4% of patients treated locally had a favourable outcome (well-functioning implant – 53.1% or patient outliving implant – 41.2%) versus 92% of patients treated at the hub hospital (well-functioning implant – 58% or patient outliving implant – 34%). Likewise, cumulative mortality rates for the two sites were comparable and reflected the fragility of this patient cohort.

## Discussion

The burden of periprosthetic fractures increases, with more successful primary arthroplasty procedures being performed and patients are living longer. The management of periprosthetic fractures is complex and mandates specialist input. The hub and spoke model are designed to ensure that patients are reviewed by the right specialist with the required skills, access to the necessary equipment/services and in an appropriate time frame.

This study provided insight into the experiences of a spoke unit following the introduction of the hub and spoke model for periprosthetic management. The limitations of this study include missing data sets pertaining to the timing of surgery and the lack of standardised outcomes recording and reporting. The success of the surgery was based on radiological parameters and the need for revision surgery. However, the patient reported outcome measures are not routinely collected for patients with a periprosthetic fracture at our institutions. As a result, the authors cannot decisively comment on the success of treatment.

The annual burden of periprosthetic fractures increased 300% over the study period. Looking forward, there needs to be appropriate financial and resource planning to support both hubs and spoke units to meet complex care needs. With advanced age and high ASA grades reflecting the fragility of this cohort, mortality rates are high. This reinforces the importance of getting it right the first time, with appropriate and timely investigation and management optimising outcomes [12]. Priorities include minimising complications, revision procedures, unnecessary patient transfers and length of hospital stay.

Seventy five percent of patients presenting to the local spoke hospital with periprosthetic fracture were managed in house. Discussion between specialists at the hub and spoke hospitals reduced patient transfer by 14%. Of the 50 patients transferred to the hub, 4% were managed conservatively. If these patients had been identified before the transfer, the transfer could have been avoided. This highlights the importance of regular joint meetings between spoke and hub staff and freedom of information transfer.

The mean patient age was higher for those managed locally (79.9 versus 75.5 yrs., *p* = 0.008). This could reflect those older, frailer patients are more likely to be managed conservatively. A review of fracture classification identifies that the majority of types A, B1, C, and D were managed locally. This may be explained by the fact that these fractures are more likely to be managed conservatively or with open reduction internal fixation, therefore within local capabilities. Most cases referred to the hub were type B2, wherein the implant is loose. These cases are more likely to require revision arthroplasty, which has been reflected in the high rate of revision arthroplasties performed on patients transferred to the hub. The authors conclude that the variation in treatment modalities between spoke and hub sites reflects the increased requirement of transferred patients and therefore confirms appropriate referral.

A key finding is that a decision to transfer a patient to the hub results in a significant delay in definitive management (4.8 versus 12 days). It is well recognised that a delay in hip fracture surgery over 24 h is associated with increased mortality, morbidity, and length of hospital stay [13]. Research has also demonstrated that delayed surgery for periprosthetic fracture is associated with increased medical complications, reoperation, length of hospital stay, cost, and mortality [14]. While these are complex cases necessitating thorough planning, delays should be minimised. Streamlining the current process would benefit from an option to fast track these vulnerable patients for definitive care. At the current rate, 75% of periprosthetic fractures are managed locally. If this is to be maintained, spoke units must be encouraged to preserve the necessary skill mix. As orthopaedic surgeons become ever more sub-specialised, there is a risk that the necessary skills be centralised to hub units, which may delay definitive management and adversely impact patient outcomes.

The limitations of this study include missing data sets pertaining to the timing of surgery and the lack of standardised outcomes recording and reporting. The success of the surgery was based on radiological parameters and the need for revision surgery. However, patient-reported outcome measures are not routinely collected for patients with a periprosthetic fracture at our institutions. As a result, the authors cannot decisively comment on the success of treatment.

## Conclusion

The current hub and spoke set-up are functioning well, with most periprosthetic fractures managed locally and with a good radiological outcome. Patient transfers to the hub unit proved appropriate in the majority of cases. However, patient transfer was associated with a significant delay in definitive treatment. For spoke management of periprosthetic fractures to be maintained, departments must look to preserve the necessary case-mix in the face of increasing sub-specialisation. To improve patient morbidity and mortality communications, pathways and protocols need to be clear and effective to minimise delay in definitive care. Patient-reported outcome measures should be standardised, collected and reported regularly to confirm the benefit of current practice.

## Conflict of interest

All authors declare no conflict of interest.

## Funding

This research did not receive any funding.

## Ethical approval

Ethical approval is not required.

## Informed consent

This article does not contain any studies involving human subjects.

## Authors contributions

SM – conceptualisation, data collection, methodology.

LH – writing original draft.

QC – Supervision, reviewing and editing.

AB – Supervision, reviewing, and editing.
